# Base Flipping in Tn*10* Transposition: An Active Flip and Capture Mechanism

**DOI:** 10.1371/journal.pone.0006201

**Published:** 2009-07-10

**Authors:** Julien Bischerour, Ronald Chalmers

**Affiliations:** University of Nottingham, School of Biomedical Sciences, The Medical School, Queens Medical Centre (QMC), Nottingham, United Kingdom; University of Minnesota, United States of America

## Abstract

The bacterial Tn*5* and Tn*10* transposases have a single active site that cuts both strands of DNA at their respective transposon ends. This is achieved using a hairpin intermediate that requires the DNA to change conformation during the reaction. In Tn*5* these changes are controlled in part by a flipped nucleoside that is stacked on a tryptophan residue in a hydrophobic pocket of the transposase. Here we have investigated the base flipping mechanism in *Tn10* transposition. As in Tn*5* transposition, we find that base flipping takes place after the first nick and is required for efficient hairpin formation and resolution. Experiments with an abasic substrate show that the role of base flipping in hairpin formation is to remove the base from the DNA helix. Specific interactions between the flipped base and the stacking tryptophan residue are required for hairpin resolution later in the reaction. We show that base flipping in Tn*10* transposition is not a passive reaction in which a spontaneously flipped base is captured and retained by the protein. Rather, it is driven in part by a methionine probe residue that helps to force the flipped base from the base stack. Overall, it appears that base flipping in Tn*10* transposition is similar to that in Tn*5* transposition.

## Introduction

**Figure 1:**
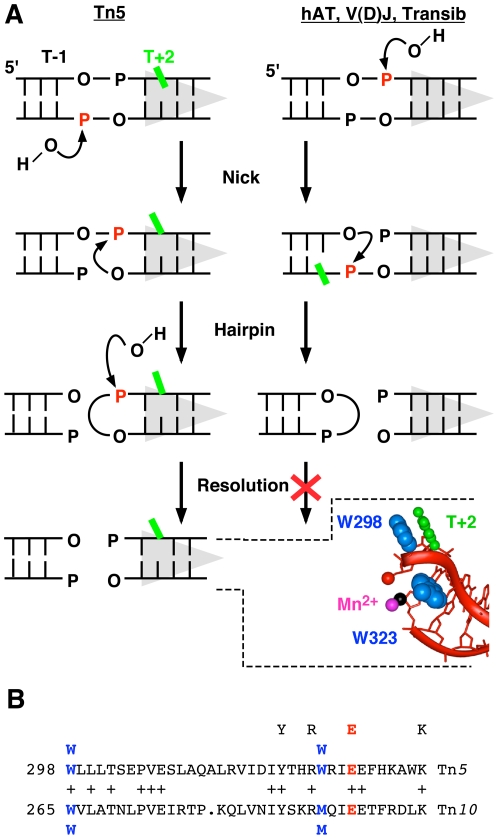
The hairpin and base flipping stages of transposition in different families of transposons. A Different families of DDE transposons have hairpin intermediates of opposite polarity. Scissile phosphates are shown in red; transposon end and RSS, grey triangles. Left panel: Binding of Tn*5* transposase creates a distortion in the DNA that destabilizes stacking of the T+2 base (green). The first step of the reaction is a nick to expose the 3′-OH at the end of the transposon. This facilitates flipping of the T+2 base from the helix in preparation for cleavage of the second strand by a direct transesterification reaction, generating a hairpin intermediate on the transposon end [35], [36]. Subsequently, the hairpin is resolved to yield a blunt transposon end. The insert shows the co-crystal structure of the Tn*5* transposon end, with the flipped base at position +2 [1]. All of the residues of the bound transposase have been omitted except for two tryptophan residues. One acts as a probe inserted into the DNA helix, while the other provides stacking interactions to stabilize the flipped base. Right panel: In the hAT transposons and V(D)J recombination the polarity of the reaction is reversed. The first nick occurs on the top strand generating a 3′-OH on the flanking DNA end [2], [37], [38]. Transesterification yields a hairpin on the flanking DNA that is processed by the host. Residue -1 on the bottom strand is distorted and becomes permanganate sensitive after the first nick (green) [17]. B The amino acid sequence of the Tn*5* transposase in the vicinity of the probe and stacking residues is aligned with the equivalent region from Tn*10* transposase. The Tn*5* transposase stacking tryptophan is at position 298 and a tryptophan occupies the equivalent position in Tn*10* transposase. However, the Tn*5* transposase probe tryptophan aligns with a methionine residue in the Tn*10* transposase. The E residue of the YREK motif is also a member of the DDE triad of residues that coordinate the catalytic metal ions and are essential for catalysis.

Mobile DNA sequences have had a profound influence on evolution. Although not as numerous as retrotransposons in higher eukaryotes, cut-and-paste DNA-transposons are a successful group of elements, well represented in all branches of life. They generally encode a single transposase protein with a characteristic DDE triad of amino acid residues in the active site. These ‘DDE’ enzymes belong to an ancient superfamily of proteins that share a common RNase H-like structural fold and catalyze phosphoryl transfer reactions using a two-metal-ion mechanism. Most superfamily members perform phosphoryl transfer reactions on only one strand of their respective nucleic acid substrates. The relative simplicity of this reaction is reflected in the compact structure of the catalytic core of these enzymes. In contrast, most cut and paste transposases, with the exception of the *mariner* family, use a hairpin intermediate to cleave the second strand of DNA at the transposon end (Figure 1A). In these enzymes the catalytic core is disrupted by the insertion of an extra sub-domain that in Tn*5* transposase interacts with a flipped base at the transposon end [1]–[4].

Base flipping is a recurrent theme in nucleic acid metabolism. The best known examples of enzymes that use this mechanism are DNA methylases, glycosylases, and glycosyl- and alkyl-transferases [5]–[9]. Other (more unusual) examples include pseudouridine synthase, sarcin/ricin toxins, certain restriction endonucleases and the Tus-*Ter* replication termination complex [10]–[15]. In most cases base flipping provides the enzyme with access to its substrate which is the base itself. The flipped base at the Tn*5* transposon end, first observed in the crystal structure of the transpososome [1], was therefore unexpected because it is not subject to any kind of modification such as methylation or excision. Nevertheless, the powerful image of the flipped base stacked on a tryptophan residue seemed to provide an explanation as to how the enzyme achieved the steric freedom required for formation of the hairpin intermediate (Figure 1A). However, biochemical experiments later showed that although the hairpin step required removal of the base from the helix, specific base stacking on the tryptophan residue occurred only later, during hairpin resolution and target site integration at the target site [16], [17].

Amongst transposons, Tn*10* and Tn*5* are considered to be close relatives even though the transposases share only 15–20% amino acid sequence identity [18]. The proteins have identical active site residues including the YREK motif, the DDE catalytic triad and the tryptophan residue that stacks with the flipped base (Figure 1B). Many base flipping enzymes also have a wedge or probe residue that intercalates into the helix, filling the space vacated by the flipped base. This can be a passive mechanism, as in DNA uracil glycosylase, where the intercalating residue moves into the space vacated when other elements of the protein capture a flipped base [19], [20]. Or the probe can actively push the flipped base from the base stack, as in the HhaI methylase [21]. In Tn*5* transposase a second tryptophan is used as the probe residue, but this is substituted by a methionine residue in Tn*10* transposase (Figure 1A, B).

Here we investigate the mechanism of base flipping in Tn*10* transposition using biochemical tools and active site mutations. We find that the mechanism is similar to *Tn5* transposition. However, Tn*10* transposition is more amenable to the permanganate base flipping assay than *Tn5* transposition. This difference allowed us to measure the separate contributions to base flipping of the probe and stacking residues. We conclude that base flipping is a central player in the choreography of the phosphoryl transfer reactions that make and resolve the hairpin intermediate.

## Results

### Base flipping increases after the first nick

Potassium permanganate reacts with thymine bases in distorted DNA, particularly if they are in an extra-helical location [22], [23]. The reagent attacks from above the plane of the base and is therefore used as a probe for base-stacking interactions. In conjunction with analysis of active site mutations, permanganate footprinting of the Tn*5* cleavage intermediates revealed that base flipping increases greatly after the first nick [17]. However, distortion of the helix was detected even before catalysis as evident from the permanganate sensitivity of the transposon end in the uncleaved transpososome complex.

**Figure 2:**
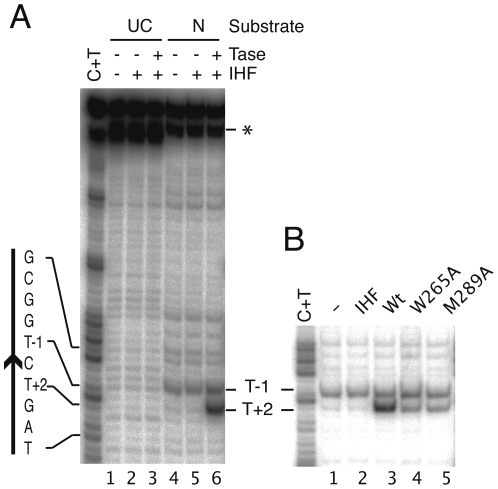
Base flipping after the first nick. Transpososomes were assembled and treated with KMnO_4_. KMnO_4_ oxidizes thymine bases in distorted DNA, particularly if they are in an extra-helical position [23]. Oxidation converts the thymine to cis-thymine glycol which, upon piperidine treatment, undergoes further degradation leading to cleavage of the DNA strand. After quenching the DNA was analyzed on a DNA sequencing gel. A The substrate was either uncleaved or pre-nicked. The nucleotide sequence of the transposon end is given on the left, with the arrowhead indicating the location of the transposon end. UC, uncleaved substrate; N, pre-nicked substrate, T'ase, transposase; IHF, integration host factor; *, This band is an artifact that appears to be caused by a heterogeneity at the 5′-end of the DNA strand. Since the label is located at the 3′-end of the DNA strand, this artifact does not contribute to the sequencing ladder or the permanganate footprints. B As in part A except that the transpososomes were assembled on the pre-nicked substrate with the wild type and mutant proteins.

To investigate whether base flipping in Tn*10* transposition also takes place after the first nick we assembled transpososome complexes with uncleaved and pre-nicked substrates and treated them with permanganate (Figure 2A). No transposase-dependent permanganate sensitivity was detected with the uncleaved transposon end. In the corresponding Tn*5* complex, residues T+2 and T-1 were slightly sensitive, indicating the early distortion of the DNA [17]. The absence of this signal in the Tn*10* transposase complex may be due to the lower efficiency of transpososome assembly, which is often as low as 5–10% and may therefore obscure a faint signal. In contrast, when the transpososome was assembled with the pre-nicked transposon end, the T+2 residue was sensitive to permanganate oxidation (Figure 2A). This indicates a significant change in the conformation of the T+2 base at the end of Tn*10*, and that it is probably flipped from the helix like its counterpart in the Tn*5* transposon end.

In Tn*5*, the flipped base at position T+2 remains protected from permanganate oxidation by its location within a hydrophobic binding pocket and/or stacking against the tryptophan residue [1], [17]. Permanganate sensitivity of the flipped base was revealed only after mutation of the stacking tryptophan residue [17]. The permanganate sensitivity of the T+2 base in the wild type Tn*10* transpososome indicates that the binding pocket in which the flipped base is putatively held is more accessible than the equivalent region in the Tn*5* transposase. This provides a technical advantage because the permanganate sensitivity assay can be used to observe Tn*10* base flipping dynamics in the wild type background.

### Probe and stacking residues both drive base flipping

Base flipping has been observed in many different enzymes. Even though many of these are unrelated, they often have mechanistic similarities. For example, base flipping is usually driven by some combination of DNA bending, the intrusion of a probe residue and stabilization of the flipped base in an extra-helical location. We therefore investigated the effects of the putative probe and stacking residue mutations on the permanganate sensitivity at base T+2 of the Tn*10* transposon end. Transpososome complexes were assembled with the wild type and mutant proteins and treated with permanganate (Figure 2B). As before, wild type transposase produced a strong permanganate signal at T+2. However, the signal was reduced by 70% and 55% in the W265A (stacking) and M289A (probe) mutants, respectively. This reveals that the probe residue has an active role in base flipping, and suggests that base flipping is driven by more than the interactions between T+2 and the stacking tryptophan.

### T+2 crosslinking increases after the first nick

The permanganate sensitivity of the flipped base in the Tn*10* transposon end suggested that the hydrophobic pocket in which it is presumably held is more accessible than the equivalent region in the Tn*5* transposase complex (above). In the Tn*5* reaction the sequestration of the flipped base is reflected in the fact that it can be photo-crosslinked to the stacking-tryptophan. Indeed, it was crosslinking that provided a firm experimental link between the flipped base observed in the cocrystal structure and the permanganate signal [17].

**Figure 3:**
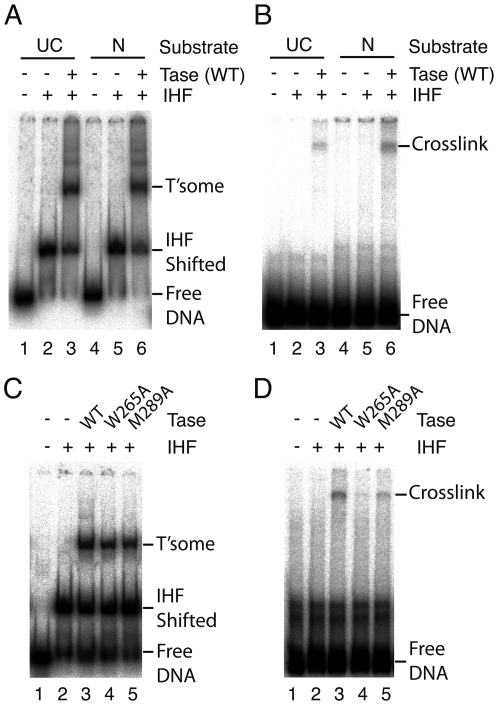
Crosslinking between transposase and the flipped base. The thymidine residue T+2 on the top strand of the DNA substrate was substituted with the zero-length crosslinking reagent iodouracil which reacts primarily with aromatic amino acid side chains. Transpososomes were assembled on 5′-end labeled uncleaved (UC) and/or pre-nicked (N) substrates. An aliquot was removed to monitor transpososome assembly by EMSA. The remainder was exposed to UV light and analyzed by SDS-PAGE. Autoradiograms are shown. A Transpososome assembly with wild type transposase was monitored by EMSA. As expected, assembly on linear DNA fragments requires the presence of the host protein IHF [29]. B UV crosslinking of the reaction mixtures from part A and analysis by SDS-PAGE. Crosslinking was detected only in the presence of transposase and was more prominent after the first nick. C and D The same as parts A and B respectively, except that the transpososomes were assembled on the pre-nicked substrate using wild type and mutant transposase.

To investigate the contacts with the putative stacking-tryptophan in the Tn*10* transposase we substituted the T+2 residue with IdU and assembled complexes using uncleaved and pre-nicked transposon ends (Figure 3A). The complexes were exposed to UV, and analyzed by SDS-PAGE (Figure 3B). With the uncleaved transposon end a faint transposase-dependent product was detected (lane 3). This signal increased 2-fold with the pre-nicked transposon end (lane 6). Under similar experimental conditions with the Tn*5* transposase crosslinking increased 5-fold [17].

The high efficiency of crosslinking in the Tn*5* reaction allowed peptide sequencing to identify the crosslinked residue [17]. However, we repeatedly failed to generate peptide sequences from Tn*10* transposase because of the low concentration of the tryptic peptides and the high background of un-crosslinked DNA. To address the lack of a rigorous experimental control, we tested the W265A stacking-residue mutant for crosslinking with the pre-nicked substrate (Figure 3C, D). The mutation largely abolished crosslinking, providing evidence, albeit indirect, that this residue is the crosslinking target.

We also tested crosslinking of the M289A probe-residue mutant to the flipped base (Figure 3D). This mutation caused a 60% reduction in crosslinking, comparable in magnitude to its effect on the permanganate sensitivity of T+2 (Figure 2B). This correlation supports our view that T+2 permanganate sensitivity and crosslinking are both good indicators of base flipping.

### The catalytic effects of probe and stacking residue mutations

**Figure 4:**
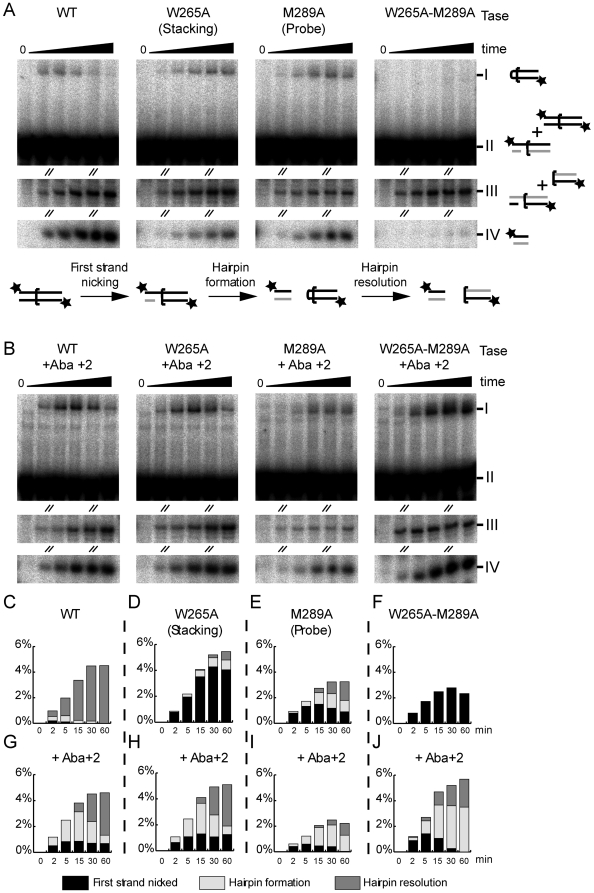
Cleavage reactions with transposase mutants and an abasic substrate. Transpososomes were first assembled in the absence of divalent metal ions. The cleavage reaction was initiated by the addition of MgCl_2_ at time zero. Aliquots were withdrawn at the indicated times and the reaction halted by the addition of EDTA and SDS. The products were analyzed on a DNA sequencing gel and recorded and quantified by autoradiography on a phosphoimager. The DNA substrates were labeled at both 5′-ends so that all three phosphoryl transfer reactions could be observed in a single experiment. The steps of the cleavage reaction are shown in panel A of the figure below the gel panel. The flanking DNA is to the left and the transposon arm to the right of the half bracket that indicates the location of the transposon end. The positions of the radioactive labels are indicated by the asterisks. Since the reactions are analyzed on denaturing gels, the unlabeled DNA strands, illustrated in grey, are not detected in the autoradiograms. The identity of each band is indicated to the right of the gel in panel A. Bands I and IV each represent a single product of the reaction as indicated. Bands II and III each represent mixtures of more than one co-migrating product and/or substrate as indicated. A & B Cleavage reactions of wild type and abasic DNA substrates. The diagonal slashes indicate regions of the gels that have been removed because they contain no relevant information. Unaltered images of the gels are provided in Figure S1. The identity of the products are indicated next to each band: Band I is the hairpin intermediate; Band II consists the unreacted substrate plus the top strand of the nicked product; Band III contains the bottom strand of the nicked product and the bottom strand of the cleaved transposon end (the resolved hairpin); Band IV contains the top strand of the cleaved flanking DNA that is released upon hairpin formation. In panel B the substrate has an abasic residue at position +2 of the top strand. This was prepared by incorporating a uracil residue at that position by PCR and subsequently treating the substrate with uracil glycosylase. This approach was preferred over one in which the abasic site could have been incorporated during oligonucleotide synthesis. Tn*10* transposon arms are folded during assembly of the transpososome [32], [39], [40], and the DNA fragments required are too long for convenient oligonucleotide synthesis. C-F Quantification of cleavage intermediates. The respective products are plotted as a percentage of the total substrate in the reaction. The amount of each intermediate present at each time point is indicated by the shading within the column. None of the conditions tested severely inhibit the nicking step of the reaction. Sixty minutes is sufficient time for all of the transpososome complexes present at the start of the reaction to achieve the first nick. The height of the column at the 60 minute time point is therefore equivalent to the efficiency of transposome assembly, which varied over a 3-fold range in the reactions presented in this experiment. Bands I and IV (corresponding to the hairpin and cleaved top strand, respectively) are unique and unambiguous products of the reaction and can be quantified directly from the gel by phosphorimager analysis. Other intermediates and/or substrate comigrate and therefore can not be quantified directly. They were calculated as follows: first strand cleavage (first nick)  =  Band III - (Band IV - Band I). Hairpin resolution  =  Band IV - Band I. These calculations rely on equal labeling efficiency at either end of the substrate. To determine the efficiency of labeling an aliquot of the substrate was cleaved into two parts by NdeI, and the ratio of label incorporated at each end of the fragment was determined by phosphoimager analysis. This ratio was used to adjust all quantifications described above.

The Tn*10* transposase probe and stacking residues were altered to alanine and the purified transposases were assayed for cleavage activity (Figure 4A). The DNA substrate was labeled at both 5′-ends so that both top and bottom strand nicks and the hairpin intermediate could be observed in a single experiment. The three phosphoryl transfer reactions that constitute cleavage of the transposon end were quantified and plotted as a percentage of the total substrate in the reaction (Figure 4C-F).

With the wild type Tn*10* transposase, neither the nicked nor hairpin intermediates accumulated as they were converted rapidly to the cleaved-end product (Figure 4A, C). With the W265A stacking residue mutation first strand nicking was normal, but the rate of hairpin formation was greatly reduced (Figure 4D). Furthermore, the accumulation of hairpin at later time-points suggests that the hairpin resolution step is slightly slow in the absence of the stacking residue. This is similar to the behavior of the equivalent Tn*5* transposase mutation [16].

The M289A probe residue mutation did not have much effect on the first nick which appeared rapidly at the start of the reaction (Figure 4A, E). However, the hairpin intermediate appeared late and persisted during the remainder of the reaction. The probe residue is therefore important for the hairpin formation and resolution steps of the reaction. The equivalent Tn*5* transposase mutant functions similarly although the hairpin resolution defect is worse (90% of the hairpin product remained after 120 minutes) [17]. Finally, the Tn*10* double mutant was completely defective for the hairpin formation step, although the kinetics of the nicking step were relatively normal (Figure 4A, F).

### T+2 interactions promote hairpin resolution

To investigate the role of the flipped base at different stages of the reaction, an abasic residue was introduced at position +2. We refer to this substrate as Aba+2. In transposition reactions with the wild type transposase, the abasic substrate underwent nicking at a similar rate to the equivalent wild type substrate (Figure 4G, compare with C). A key difference between the wild type and abasic substrates was observed at the hairpin intermediate stage, where the hairpin accumulated because it was not resolved efficiently (Figure 4G). This suggests that direct interactions between the protein and the flipped base are important for hairpin resolution. This is consistent with the phenotype of the Tn*10* W265A mutant on the unmodified DNA substrate where hairpin resolution is also somewhat impaired (Figure 4D).

### Unsequestered T+2 interferes with hairpin formation

It has already been shown that the stacking residue mutation inhibits hairpin formation on the normal Tn*10* transposase substrate (Figure 4D). When this mutant was assayed with the Aba+2 substrate, the hairpin defect was completely rescued (Figure 4H). Hairpin resolution was slow compared to the fully wild type situation, but no more so than with the wild type protein on the Aba+2 substrate (compare Figure 4G and H). This suggests that the hairpin-formation defect of the W265A mutant is caused by interference from the T+2 residue which is no longer retained in its proper extra-helical location by the stacking interaction.

We next tested whether the M289A mutation could be rescued by the Aba+2 substrate (Figure 4B, I). The kinetics of the first nick were largely unchanged. However, the amount of hairpin intermediate present at each time point was greater than in the equivalent wild type situation. It therefore appears that with the Aba+2 substrate the hairpin is produced more quickly but resolved more slowly compared to the wild type substrate (compare Figure 4 E and I). The behavior of the double mutant on the Aba+2 substrate was very similar (compare Figure 4F and J). Together these results show that T+2 interferes with hairpin formation, but promotes hairpin resolution.

## Discussion

A general model has been proposed for base flipping reactions [24]. Protein binding first distorts the phosphodiester backbone to provide an exit route for the target base out of the helix. A probe residue pushes the base out of the helix and/or fills the space vacated. Finally, the flipped base is trapped in an extra-helical location by stabilizing interactions with the protein. Our previous work revealed that Tn*5* transposase uses all three of these mechanisms. However, the role of the probe residue remained ambiguous, particularly whether it is actively involved in base flipping, or serves only to fill the space vacated by the flipped base.

### An active flip and capture mechanism in Tn*10* transposition

Our present results suggest that the mechanism of base flipping in Tn*10* transposition is similar to Tn*5* transposition [16], [17]. Base flipping in Tn*5* transposition could not be detected directly using the permanganate assay because the base was protected in a hydrophobic binding-pocket in the protein. Base flipping was only revealed after the protective pocket was made more accessible by mutation of the stacking tryptophan residue [17]. In contrast, the equivalent region in the Tn*10* transposase appears to be more accessible, and base flipping can be detected by the permanganate sensitivity assay with the wild type protein. This made it possible to determine the separate contributions of the probe and stacking residues in an otherwise wild type background (Figure 2).

The stacking residue mutation (W265A) reduced permanganate sensitivity by 70%, showing that this residue contributes substantially to maintenance of the flipped state (Figure 2B). The probe residue mutation (M289A) reduced permanganate sensitivity by 55%, suggesting that it is not merely moving in to fill the space vacated by the flipped base. Rather, it suggests that the probe may help force the base from the base stack. However, the present results do not provide conclusive proof of this view which would require initial rate measurement, for example, by proton exchange.

The probe and stacking residues are important for hairpin formation and resolution. This is evidenced by the accumulation of nicked and hairpin products in cleavage reactions with the respective mutant transposases (Figure 4D, E ). The Aba+2 substrate rescues hairpin formation, but not resolution (Figure 4H, I ). This suggests that hairpin formation requires the absence of the T+2 residue from the helix. In contrast, efficient hairpin resolution requires the interaction between the flipped T+2 residue and the stacking tryptophan. This is similar to what occurs in Tn*5* transposition. In this case we previously suggested that the flipped base could be likened to a handle that is used by the protein to position the second strand of DNA in the active site [17].

The probe residue also appears to have a role in hairpin resolution as this intermediate accumulates in cleavage reactions with the M289A transposase (Figure 4E). This may be an indirect consequence of the base flipping defect in this mutant which would reduce the crucial interaction between the flipped base and the stacking tryptophan. However, the probe residue may also have a direct role in positioning the DNA strand during hairpin resolution.

### Base flipping and the reversed hairpin polarity of eukaryotic transposons

The RAG1/2 V(D)J recombinase is one of the eukaryotic members of the DDE family in which the polarity of the hairpin intermediate is reversed. The reversed polarity of the hairpin intermediate begs the question as to whether any elements of the Tn*5*/Tn*10* base flipping mechanism are conserved in these enzymes [3], [25]. Our own investigation of V(D)J recombination, using a similar strategy to that applied here, demonstrated that although the base at position -1 is apparently flipped, it does not appear to engage in a specific interaction with aromatic amino acid residues in the recombinase that would indicate capture of the flipped base (JB and RC to be presented elsewhere).

Our current results with Tn*10* indicate that efficient hairpin resolution depends on the interaction of the flipped base with tryptophan 265. The eukaryotic members of the DDE family such as RAG1, which do not resolve the hairpin intermediate, are therefore perhaps unlikely to capture the flipped base in a highly specific interaction, especially since it is probably located in the flanking DNA where the sequences are in any case variable.

## Materials and Methods

### Proteins

Wild type Tn*10* transposase was expressed from pRC60 which contained the transposase gene on an NdeI-BamHI fragment cloned into pET11a. The W265A and M289A mutations were introduced by site directed mutagenesis. The proteins were expressed and purified as described in [26]. The host protein integration host factor (IHF) was expressed from pRC188, which identical to pPR204 obtained from Phoebe Rice. This plasmid contains the himA and hipD genes encoding IHF subunits cloned as an operon in pET27b. The protein was expressed and purified essentially as described in [27], [28]. IHF is require to fold the linear DNA fragments during assembly of the Tn*10* transpososome [29], [30]. A MonoS chromatography step was added to the purification procedure. The column buffer was 25 mM Tris pH 8, 1 mM EDTA, 2 mM DTT and 10% glycerol. Elution was with a 20 ml gradient from 50 mM to 2 M NaCl in the same buffer. Pooled fractions were stored at −80°C. The nicking endonuclease N.BsaI was a gift from Shuang-yong Xu at New England Biolabs [31].

### Oligonucleotides and plasmid construction

The following oligonucleotides were used: *Tn10Armshort*, 5′CGAGGTCGACCCGAAACCATTTG; *NBsaExt*, 5′GGTCTAGGTGAGCGTGGGTCTCGCGGTCTGATGAATCCCCTAATGATTTTGG; *UCTn10*, 5′GGTCTAGAGTGAGCGTGGGTCTCGCGGTCTG; *UCIdUTn10*, 5′GGTCTAGAGTGAGCGTGGGTCTCGCGGTC{IdU}G; *UCdUTn10*, 5′GGTCTAGAGTGAGCGTGGGTCTCGCGGTC{U}G. Modified residues appear within curly brackets. The uracil was used for generating the abasic substrate and the iodo uracil was for protein-DNA crosslinking. The plasmid pRC915 (pJB15) was constructed as follows. The outside end of IS*10*-Right was amplified from pRC98 (pKC3) [32] using oligonucleotides Tn10Armshort and NBsaExt. The fragment was cloned into pDRIVE (Qiagen) from which the ampicillin gene had been removed to yield a plasmid with a single BsaI recognition site overlapping the transposon end. This site was used to generate the pre-nicked substrate by digestion with N.BsaI.

### DNA substrates

Linear transposon ends for the permanganate assay were generated by digesting pRC915 with EcoRI and AccI. This yielded a fragment with 83 bp of transposon and 47 bp of flanking DNA sequences. If a pre-nicked transposon end was required the plasmid was previously treated with the nicking endonuclease N.BsaI at 50°C for 4 h. The DNA fragments were 3′-end labeled with ^32^P using dCTP and the exo^-^ Klenow fragment (NEB). This added a single ^32^P cytosine residue to the top strand of the transposon arm. The opposite strand remained unlabeled. The labeled transposon end fragment was purified by electrophoresis in a TBE-buffered 5% polyacrylamide gel, and recovered by the crush and soak method as described [29], [33].

Linear transposon ends for the cleavage assays were generated by PCR using the primers Tn10Armshort and UCTn10 with plasmid pRC915 as template. This yielded a fragment with 91 bp of transposon and 28 bp of flanking DNA sequences.

The DNA fragment with the IdU crosslinking reagent at position +2 of the transposon end was generated using the primer pair Tn10Armshort and UCIdUTn10. This yielded a fragment with 91 bp of transposon and 28 bp of flanking DNA sequences.

The DNA fragment used for preparing the transposon arm with an abasic site at position +2 was generated using the primer pair Tn10Armshort and UCdUTn10. The PCR product was treated with uracil DNA glycosylase (NEB) for 2 h. The abasic site was stabilized by making the solution 100 mM in freshly diluted NaBH_4_ and incubating on ice for 30 min. The DNA was then purified using a MicroSpin G-50 gel filtration device (Amersham Pharmacia).

The PCR generated substrates were end labeled with ^32^P using T4 polynucleotide kinase, and purified as described above for the 3′-end labeled substrates.

### Transpososome assembly and cleavage assays

Transpososome assembly mixtures and quantification of the complexes by EMSA was as previously described [29], [34] except that the Tris buffer was replaced by Hepes pH 7.5 and DTT was omitted from all solutions. Standard reactions (20 µl) contained 2 nM radiolabeled substrate, 10 nM IHF and approximately 1 nM transposase. The efficiency of Tn*10* transpososome assembly is variable from day-to-day and we routinely performed protein titrations which were used to normalize the level of complex formation with the different proteins and substrates. For the permanganate and crosslinking assays the reactions also contained 4 mM CaCl_2_. Transposase was added last and the mixture was incubated at room temperature for 1.5 h. The cleavage reaction was initiated by making the mixture 5 mM in MgCl_2_.

### Crosslinking assay

Transpososomes were assembled as described above with the IdU substrate. An aliquot was analyzed by EMSA to determine the efficiency of assembly. Half of the remaining material was exposed to a Stratagene 2040 EV transilluminator (five 312 nm fluorescent tubes each rated at 15 W power) for 40 min from above at a distance of 8 cm. The other half of the sample was retained as an unexposed control. Samples were mixed with Laemmli SDS-PAGE loading buffer, heated for 20 min at 50°C and then analyzed by Laemmli SDS-PAGE using 8% polyacrylamide. The gels were dried and quantified with a Fuji Phosphorimager.

### Permanganate base flipping assay

Complexes were assembled and treated with 8 mM KMnO_4_ for 30 S at room temperature before quenching with a ‘stop solution’ that achieved a final concentrations of 300 mM NaOAc pH 5.5, 30 mM EDTA, 100 mM DTT and 100 µg/ml glycogen. The DNA was recovered in a pellet by ethanol precipitation. The pellet was dissolved in 100 µl of freshly diluted 1M piperidine by heating to 90 °C for 30 min. Following a second round of ethanol precipitation the DNA was dissolved in TE buffer plus 7 M urea and analyzed on a 10% polyacrylamide DNA sequencing gel. The products were identified by reference to a Maxam-Gilbert C+T sequencing ladder generated by treating the substrate with 60% hydrazine for 10 min before quenching as described above.

## Supporting Information

Figure S1 (8.12 MB TIF)
