# Supplementary figures and images for: Base Flipping in Tn10 Transposition: An Active Flip and Capture Mechanism
Source: PLoS One. 2009 Jul 10;4(7):e6201. doi: 10.1371/journal.pone.0006201 (PMC2705183; doi:10.1371/journal.pone.0006201)

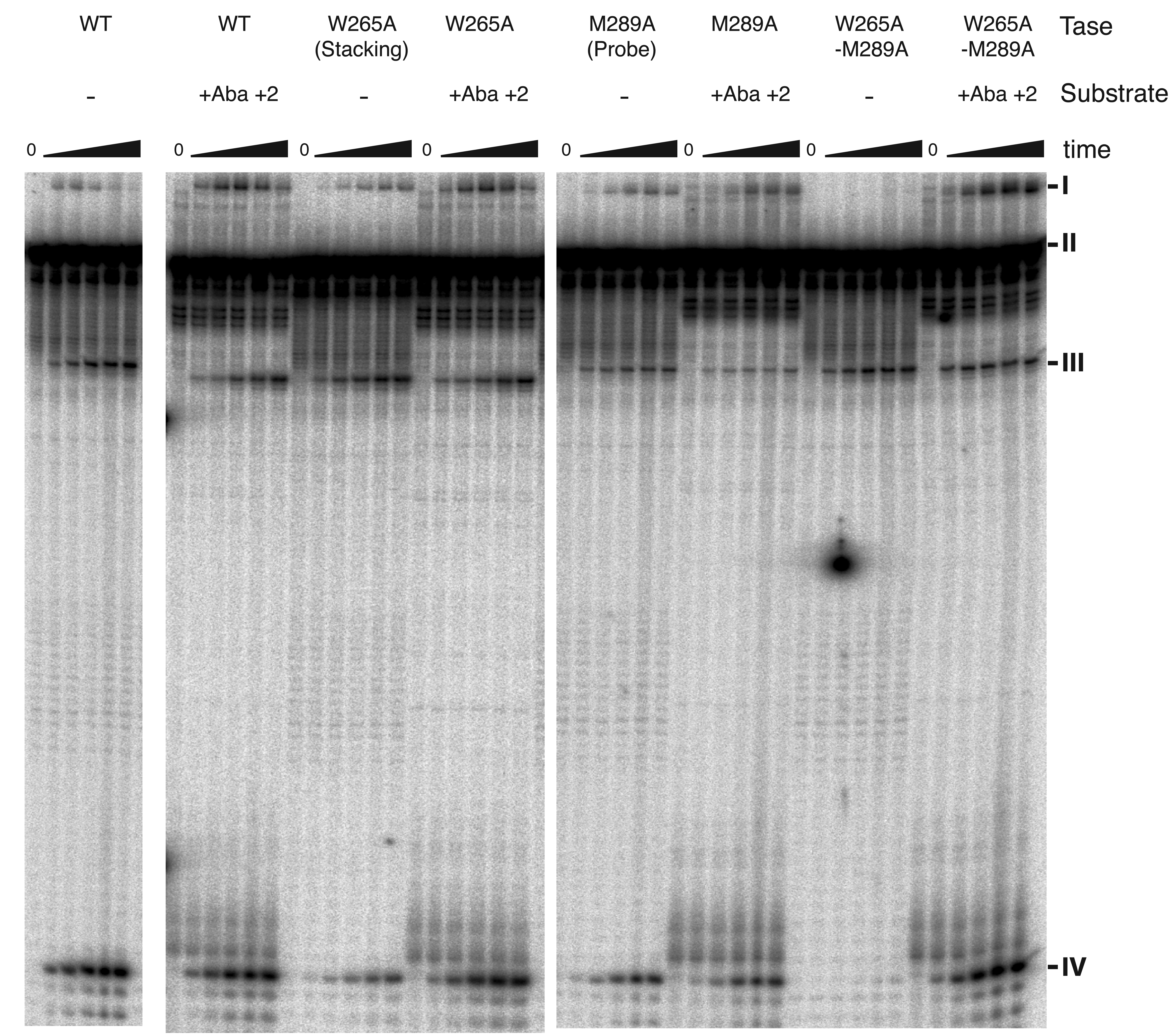

Supplement: Figure S1 — (8.12 MB TIF) [file pone.0006201.s001.tif]
